# Effects of Exercise on AMPK Signaling and Downstream Components to PI3K in Rat with Type 2 Diabetes

**DOI:** 10.1371/journal.pone.0051709

**Published:** 2012-12-13

**Authors:** Shicheng Cao, Bowen Li, Xuejie Yi, Bo Chang, Beibei Zhu, Zhenzhen Lian, Zhaoran Zhang, Gang Zhao, Huili Liu, He Zhang

**Affiliations:** 1 Department of Sport Medicine, College of Basic Medical Sciences, China Medical University, Shenyang, Liaoning Province, China; 2 Department of Exercise Science, Shenyang Sport University, Shenyang, Liaoning Province, China; 3 Department of Medicine, Division of Endocrinology, and the Barnstable Brown Diabetes and Obesity Center, University of Kentucky, Lexington, Kentucky, United States of America; Virgen Macarena University Hospital, School of Medicine, Spain

## Abstract

Exercise can increase skeletal muscle sensitivity to insulin, improve insulin resistance and regulate glucose homeostasis in rat models of type 2 diabetes. However, the potential mechanism remains poorly understood. In this study, we established a male Sprague–Dawley rat model of type 2 diabetes, with insulin resistance and β cell dysfunction, which was induced by a high-fat diet and low-dose streptozotocin to replicate the pathogenesis and metabolic characteristics of type 2 diabetes in humans. We also investigated the possible mechanism by which chronic and acute exercise improves metabolism, and the phosphorylation and expression of components of AMP-activated protein kinase (AMPK) and downstream components of phosphatidylinositol 3-kinase (PI3K) signaling pathways in the soleus. As a result, blood glucose, triglyceride, total cholesterol, and free fatty acid were significantly increased, whereas insulin level progressively declined in diabetic rats. Interestingly, chronic and acute exercise reduced blood glucose, increased phosphorylation and expression of AMPKα1/2 and the isoforms AMPKα1 and AMPKα2, and decreased phosphorylation and expression of AMPK substrate, acetyl CoA carboxylase (ACC). Chronic exercise upregulated phosphorylation and expression of AMPK upstream kinase, LKB1. But acute exercise only increased LKB1 expression. In particular, exercise reversed the changes in protein kinase C (PKC)ζ/λ phosphorylation, and PKCζ phosphorylation and expression. Additionally, exercise also increased protein kinase B (PKB)/Akt1, Akt2 and GLUT4 expression, but AS160 protein expression was unchanged. Chronic exercise elevated Akt (Thr^308^) and (Ser^473^) and AS160 phosphorylation. Finally, we found that exercise increased peroxisome proliferator-activated receptor-γ coactivator 1 (*PGC1*) mRNA expression in the soleus of diabetic rats. These results indicate that both chronic and acute exercise influence the phosphorylation and expression of components of the AMPK and downstream to PIK3 (aPKC, Akt), and improve GLUT4 trafficking in skeletal muscle. These data help explain the mechanism how exercise regulates glucose homeostasis in diabetic rats.

## Introduction

Obesity and insulin resistance are markers of type 2 diabetes [1]. Consumption of a high-fat diet (HFD) can induce obesity, insulin resistance and hyperinsulinemia, and is a widely used animal model to study insulin resistance [2], [3], [4]. However, this model does not develop hyperglycemia, another characteristic of type 2 diabetes in humans, limiting experimental studies focusing on the effects of exercise and antidiabetic drugs on blood glucose levels [5]. Therefore, extragenetic, outbred Sprague–Dawley rats are commonly fed a HFD in combination with low-dose streptozotocin (STZ) to establish type 2 diabetes, characterized by insulin resistance and β cell dysfunction [5], [6]. This model replicates the pathogenesis and metabolic characteristics of type 2 diabetes in humans [5], [6], [7]. However, the potential mechanism involved in these changes is still poorly understood. In addition, few studies have examined whether exercise influences skeletal muscle AMPK activity, downstream components of the insulin signaling pathway, or metabolism in HFD- and low-dose STZ-induced diabetic rats.

AMPK is a fuel-sensing enzyme that senses an increase in the intracellular AMP/ATP ratio during exercise or hypoxia [8], [9]. Interestingly, animal models of insulin resistance show decreased AMPK activity in skeletal muscle [10], [11]. Changes in AMPK activity may accelerate the progression of insulin resistance and metabolic abnormalities [12]. Exercise can activate skeletal muscle AMPK, effectively resisting diet- or lipid-induced insulin resistance [13], [14]. However, in mouse models with decreased AMPK activity, inhibiting AMPK activity only weakly or did not influence muscle contraction-induced glucose absorption [15], [16]. Additionally, LKB1, the main upstream kinase of AMPK, can influence glucose transport in skeletal muscle, and skeletal muscle-specific deprivation of LKB1 was reported to partly inhibit muscle contraction-induced glucose absorption [17]. Considering the potential discrepancies in these earlier results, it is necessary to further examine the mechanism by which exercise influences the AMPK pathway for regulation of metabolism.

Insulin and exercise/muscle contraction promote intracellular glucose transporter 4 (GLUT4) translocation to membrane, increasing skeletal muscle glucose uptake [18], [19]. Chronic exercise also promotes the increase in the number of GLUT4 transporters [18]. Early studies have shown that insulin and exercise both enhance GLUT4 translocation and glucose transport through divergent signaling mechanisms. The insulin signaling pathway involves rapid phosphorylation of insulin receptor and the tyrosine residues of insulin receptor substrate-1 and -2, and activation of PI3K [20], [21]. By contrast, exercise and muscle contraction do not affect insulin receptor or insulin receptor substrate-1 phosphorylation, or PI3K activity [21], [22]. However, further studies of the insulin signaling molecules downstream to PI3K have shown that atypical protein kinase C (aPKC)ζ/λ is responsive to both insulin and exercise [23], [24]. Insulin stimulation results in aPKCζ/λ translocation to the plasma membrane in cultured muscle cells and also increases its association with GLUT4 vesicles [25]. But, no studies have confirmed the effects of exercise on aPKC expression and phosphorylation in rats with HFD- and low-dose STZ-induced diabetes.

As type 2 diabetes is common in the middle-aged and elderly population, we used 15-month-old Sprague–Dawley rats in this study, and diabetes was established by HFD feeding and low-dose STZ injection. Based on previous studies [13], [14], [26], we hypothesized that exercise would improve the phosphorylation and expression of AMPK and its signaling pathway components, as well as molecules downstream of PI3K, to ultimately enhance insulin sensitivity in skeletal muscle. Thus, the present study sought to investigate whether this model of diabetes was associated with impaired skeletal muscle AMPK and PI3K signaling pathways, and whether chronic and acute exercise could improve the phosphorylation and expression of signaling molecules involved in both pathways, particularly aPKC. The study also examined the effects of HFD on AMPK and aPKC.

## Materials and Methods

### Ethics Statement

Male Sprague–Dawley rats, weighing 450–470 g, were provided by the Laboratory Animal Center of Academy of Military Medical Sciences of the Chinese People’s Liberation Army (certification No. SCXK (army) 2007–004). Experimental procedures were performed in accordance with the Guidance Suggestions for the Care and Use of Laboratory Animals, formulated by the Ministry of Science and Technology of the People’s Republic of China and were approved by the Animal Ethics Committee of China Medical University. All surgeries were performed under anesthesia, and all efforts were made to minimize suffering. The animals were killed under anesthesia (200 mg/kg thiopental) following the recommendations of the US National Institutes of Health.

### Animals and Components of Diet

These rats were housed in standard polypropylene cages, with three animals per cage, at a temperature of (22±2)°C, humidity of (50±10)%, and 12-h light/dark cycle. The high-fat diet consisted of 23% soy protein (15% energy), 19.8% pork fat (33% energy), 19.8% corn oil (33% energy), 24.5% sucrose (20% energy), and 5% cellulose, and was supplemented with 1.4% vitamin mixture, 6.7% mineral mix, 0.2% choline bitartrate, and 0.004% dibenzyltoluene. The routine control diet contained 57.3% carbohydrate, 18.1% protein, 18.8% cellulose, and 4.5% fat [27]. The diet was provided by the Animal Center of China Medical University, China.

### Establishment of Type 2 Diabetes

The rats were randomly assigned to the following groups: control(CON), high-fat diet (HFD), high-fat diet+chronic exercise (HFD +CE), high-fat diet+streptozotocin (HFD+STZ), high-fat diet+streptozotocin+chronic exercise (HFD+STZ+CE), and high-fat diet+streptozotocin+acute exercise( HFD+STZ+AE) (8 rats per group). The CON group of rats received the routine control diet for 20 weeks, with free access to water. The HFD groups were fed with the HFD for 20 weeks, with free access to water. After 8 weeks of diet intervention, the HFD+STZ, HFD+STZ+CE, and HFD+STZ+AE groups received one intraperitoneal injection of low-dose STZ (30 mg/kg, Sigma), while other groups were injected once with citrate buffer solution (pH 4.4, 1 mL/kg). Four weeks after STZ injection, the animals were deprived of food for 12 h. Diabetes was regarded as successfully induced in rats with fasting blood glucose levels ≥7.8 mmol/L and postprandial blood glucose ≥11.1 mmol/L [5], [7]. Body mass, water intake, and food intake were determined every week.

### Chronic Exercise

The rats were trained to swim over 2 d, for 10–20 min per session, to reduce water-induced stress. Two or three rats, as one group, were placed in a plastic pool, 45 cm in diameter, 60 cm deep, and water temperature of 34–35°C. After the initial training, the rats underwent chronic exercise for 1 h/d, 5 d/wk, for 8 wk. The exercise program was conducted essentially as described by Luciano *et al* [28] with some modifications.

### Acute Exercise

The HFD+STZ+AE rats received acute exercise after 20 weeks of HFD. Acute exercise was conducted in the same conditions as for chronic exercise. Acute exercise consisted of two sessions lasting 1.5 h each, with a 45 min interval between each session. The exercise program was conducted as previously described [29] with some modifications.

### Blood Sample Collection and Blood Biochemistry

At 24–36 h after the final session of chronic exercise, or 8–16 h after acute exercise, all rats were anesthetized by an intraperitoneal injection of sodium thiopental (40 mg (kg body weight)^−1^ ). Blood samples were collected from tail veins and centrifuged at 1 100×*g* for 10 min to separate serum. Serum glucose, triglyceride, total cholesterol, and free fatty acid concentrations were determined using an autoanalyzer (RT-1904C; Rayto, Shenzhen, China). Serum insulin concentrations were determined using a radioimmunoassay, as previously described [30].

### Insulin Tolerance Test and Serum Insulin Quantification

Insulin tolerance tests were performed after blood sample collection. Briefly, the rats were fasted for 12 h, anesthetized, and administered with 1.5 IU/kg artificial insulin (Sigma). Blood samples were collected at 0, 5, 10, 15, 20, 25, and 30 min after injection, centrifuged at 1100×g, at 4°C for 15 min, and stored at –20°C to determine glucose concentrations. The rate constant for plasma glucose disappearance (*K*_itt_) was calculated using the formula 0.693/biological half life. The plasma glucose half-life (t_1/2_) was calculated from the slope of least squares analysis of the plasma glucose concentration during the linear phase of the decline [31].

### Skeletal Muscle Sampling

After the insulin tolerance test, the animals were intraperitoneally injected with sodium thiopental (200 mg/kg body weight; recommended by the US National Institutes of Health) and sacrificed. Bisected soleus was used from rat to perform glucose uptake and placed in 6 mL of Krebs–Ringer bicarbonate buffer (KRBB: 117 mmol/L NaCl, 4.7 mmol/L KCl, 2.5 mmol/L CaCl_2_, 1.2 mmol/L KH_2_PO_4_, 1.2 mmol/L MgSO_4_, 24.6 mmol/L NaHCO_3_) supplemented with 8 mmol/L D-glucose for 20 min. For insulin treatment, muscles were preincubated and then incubated in the presence of 1 µM insulin for 40 min. The buffer was continuously infused with 95% O_2_/5% CO_2_ at 37°C [32], [33]. The uptake of 3-O-methyl-D-glucose (3MG) was determined as described below. The remainders of the soleus were placed in liquid nitrogen and stored at –80°C for western blotting and real time PCR. Meanwhile, gastrocnemius muscles were isolated and stored at –80°C to measure glycogen content.

### 3MG Uptake

To detect glucose transport, the muscles were cultured in KRBB containing 1 mmol/L 3MG (1.5 µCi/mL) and 7 mmol/L D-[14C] mannitol (0.3 µCi/mL; New England Nuclear, Boston, MA, USA) at 30°C for 10 min [32], [33]. The tissue was then frozen in liquid nitrogen. The muscles were weighed, thawed in 1 mol/L NaOH (450 µL) at 80°C for 10 min, neutralized with 1 mol/L HCl, and centrifuged at 20 000×*g* for 2 min. Liquid scintillation counting was used to determine the radioactivity of double-labeled digested protein solution and to calculate 3MG uptake.

### Tissue Extraction and Western Blotting

The frozen muscles were thawed, weighed, roughly cut, placed in protein extraction solution (1% Triton X-100, 100 mM Tris, pH 7.4, containing 100 mM sodium pyrophosphate, 100 mM sodium fluoride, 10 mM ethylenediaminetetraacetic acid, 10 mM sodium vanadate, 2 mM phenylmethyl sulfonylfluoride, and 0.1 mg/mL aprotinin), and ultrasonicated at maximum speed at 4°C for 30 s (JY92-IIN; Scientz, Ningbo, China). The homogenate was centrifuged at 9 000×*g* at 4°C for 40 min (HC-3618R; Zonkia, Hefei, China). Non soluble material was discarded. The protein concentration in the supernatant was quantified using Bradford’s method. Then, 60 µg of tissue extract was mixed with an equal volume of 3× sample buffer solution (6.86 M urea, 4.29% SDS, 300 mM DTT, and 43 mM Tris·HCl, pH 6.8) at room temperature for 30 min [34], subjected to sodium dodecyl sulfate–polyacrylamide gel electrophoresis (SDS-PAGE, 10% polyacrylamide gels), and transferred to a polyvinylidene difluoride membrane at 4°C for 2 h. The membrane was blocked using trihydroxymethyl aminomethane buffer salt+Tween-20 (TBST) containing 5% bovine serum albumin (Sigma), and washed with TBST (pH 7.4). The antibody was dissolved in TBST containing 1% bovine serum albumin overnight at 4°C. The following antibodies were used: phosphorylated (p)-AMPKα1 (Thr^172^), AMPKα1, p-AMPKα2 (Thr^172^), AMPKα2, p-AMPKα1/2 (Thr^172^), AMPKα1/2, p-Akt1 (Ser^473^), Akt1, p-Akt2 (Ser^474^), Akt2, p-LKB1 (Ser^431^), LKB1, p-PKCζ/λ (Thr^410/403^), p-PKCζ (Thr^410^), PKCζ (Santa Cruz Biotechnology, Santa Cruz, CA, USA; 1∶700 dilution); p-AS160 (Thr^642^), AS160, acetyl CoA carboxylase (ACC), p-ACC (Ser^79^), p-Akt (Ser^473^), p-Akt (Thr^308^), Akt, and GLUT4 (Cell Signaling Technology, Beverly, MA, USA; 1∶1 000 dilution). Bands of interest were visualized by enhanced chemiluminescence and absorbance was determined using FluorChem V 2.0 gel imaging analysis software (Alpha Innotech, San Leandro, CA, USA).

### Skeletal Muscle Glycogen Content

The frozen muscles were weighed, digested with 1 mol/L NaOH (1∶9 wt/vol) at 80°C for 10 min, neutralized with 1 mol/L HCl, and mixed with 6 mol/L HCl to a final concentration of 2 mol/L HCl. The resulting solution was incubated at 85°C for 2 h and neutralized with 5 mol/L NaOH [35]. A glucose hexokinase assay kit (Sigma) was used to determine the concentration of hydrolyzed glucose, and glucose content was determined as µmol per 1 g tissue (wet weight).

### Real-time PCR

Total RNA was extracted from the frozen muscles using RNAiso Plus (Takara, Dalian, China), and the RNA concentration was measured at 260/280 nm. RNA samples were reverse-transcribed using a PrimeScript RT reagent kit (Takara). Real-time PCR and subsequent data were analyzed using a 7900HT Fast Real-Time PCR System (Applied Biosystems, Foster City, CA, USA). β-actin was used as an internal reference, and the mRNA expression of target genes is shown relative to β-actin. The forward and reverse primer sequences were as follows: peroxisome proliferator-activated receptor-γ coactivator (*PGC1α*), 5′-CACCGTAAATCTGCGGGATG-3′ and 5′-TATCCATTCTCAAGAGCAGCGAAAG-3′; nuclear respiratory factor 1 (*NRF1*), 5′-CACTCTGGCTGAAGCCACCTTAC-3′ and 5′-TCACGGCTTTGCTGATGGTC-3′; and β-actin, 5′-CCGTAAAGACCTCTATGCCAACA-3′ and 5′-GCTAGGAGCCAGGGCAGTAATC-3′, respectively.

### Statistical Analysis

Results are expressed as mean ± SE. Differences between the control and HFD, control and HFD+STZ, HFD and HFD+exercise, and HFD+STZ and HFD+STZ+exercise groups were compared by one-way analysis of variance. Values of *P*<0.05 were considered statistically significant. Data were analyzed using JMP software (SAS Institute, Cary, NC, USA).

## Results

### Characteristics of the Rat Model of Type 2 Diabetes and Effects of Chronic Exercise

As shown in Table 1, compared with the control group, no differences were found in body mass or epididymal fat mass in the HFD+STZ group. However, food intake, blood glucose, triglyceride, total cholesterol, and free fatty acid were increased, while *K*_itt_, 3MG uptake and glycogen content were lower in the HFD+STZ group. These results indicate that HFD+STZ induced disorders in glucose and lipid metabolism, common metabolic characteristics of diabetes, and could be used to establish a rat model of type 2 diabetes, mimicking diabetes in humans. Compared with HFD rats, food intake and blood glucose were increased, while body mass, epididymal fat, insulin, and *K*_itt_ were reduced in the HFD+STZ rats (Table 1). Meanwhile, compared with control rats, the HFD increased body mass, epididymal fat mass, blood insulin concentration, blood total cholesterol and free fatty acid concentrations, but reduced *K*_itt_, 3MG uptake and glycogen content, confirming the HFD used induced insulin resistance.

**Table 1:** Metabolic characteristics and effect of exercise on body weight and biochemical parameters.

Item	metabolic characteristics			effect of exercise	
	CON	HFD	HFD+STZ	HFD+CE	HFD+STZ+CE
**Body mass, g**	520.7±12.0	598.2±15.85^**^	502.8±10.51^††^	546.5±12.8^§^	527.3±10.77
**Epididymal fat, g**	8.83±0.43	12.05±0.60^**^	7.86±0.34^††^	10.10±0.48^§^	8.61±0.44
**Food intake, g/kg/d**	63.12±1.06	65.38±1.41	80.12±4.78^**††^	64.26±1.34	75.11±4.36
**Blood glucose, mmol/L**	5.35±0.23	5.88±0.18	13.18±0.52^**††^	5.56±0.25	11.57±0.42^‡^
**Insulin, pmol/L**	66.14±2.80	96.11±6.84^**^	76.29±4.17^†^	74.35±3.81^§^	70.86±3.61
**Triglyceride, mmol/L**	1.68±0.09	1.98±0.11	2.06±0.11^**^	1.79±0.09	1.79±0.10^‡^
**Total cholesterol, mmol/L**	4.15±0.26	6.45±0.35^**^	5.78±0.39^**^	5.46±0.24^§^	4.69±0.21^‡^
**Free fatty acid, mmol/L**	0.39±0.01	0.51±0.04^*^	0.63±0.05^**^	0.43±0.04	0.49±0.04^‡^
***K*** **_itt_, %/min**	3.19±0.19	2.66±0.15^*^	1.95±0.30^**†^	3.64±0.30^§^	2.87±0.22^‡^
**3MG uptake,µmol/g/h**	0.13±0.014	0.066±0.011^**^	0.059±0.007^**^	0.089±0.004^§^	0.079±0.004^‡^
**Muscle glycogen, µmol/g wet weight**	23.64±0.29	22.83±0.23^*^	22.50±0.23^**^	23.55±0.18^§^	23.41±0.36^‡^

Values are means ± SE.*P<0.05 and **P<0.01 *vs.* the control group; †*P*<0.05, ††*P*<0.01 and ^§^ *P*<0.05 *vs.* the HFD group;^ ‡^ *P*<0.05 *vs.* the HFD+STZ group. CON: control; HFD: high-fat diet; STZ: streptozotocin; CE: chronic exercise. N = 8.

As shown in Table 1, chronic exercise reduced blood glucose, triglyceride, total cholesterol, and free fatty acid concentrations, and increased the glucose disappearance rate, 3MG absorption and glycogen content in HFD+STZ rats. However, blood insulin concentration, body mass and epididymal fat mass remained unchanged. In addition, chronic exercise decreased body mass, epididymal fat mass, blood insulin concentration, and total cholesterol, but increased *K*_itt_, 3MG absorption and glycogen content in the HFD rats (Table 1).

### Chronic Exercise Enhanced the Phosphorylation and Expression of Protein Kinase Cascades in the AMPK Pathway

**Figure 1:**
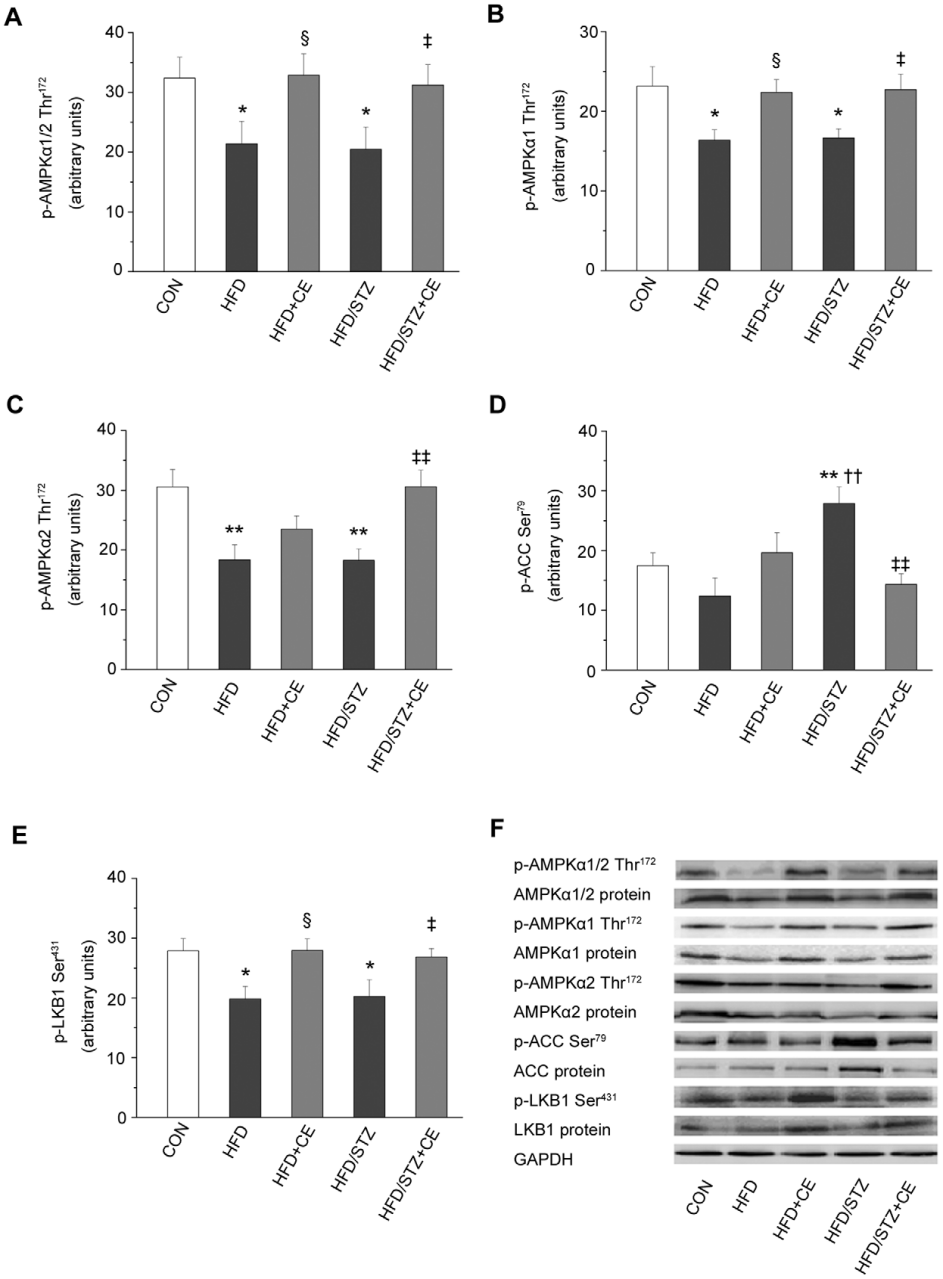
Effects of chronic exercise on the protein kinases in the AMPK pathway. A–E: relative levels of p-AMPKα1/2 (Thr^172^) (A), p-AMPKα1 (Thr^172^) (B), p-AMPKα2 (Thr^172^) (C), p-ACC (Ser^79^) (D), and p-LKB1 (Ser^431^) (E). F: representative western blots for each protein of interest. Values are means ± SE. ^*^ *P*<0.05 and ^**^ *P*<0.01 *vs.* the control group; ^§^ *P*<0.05 *vs.* the HFD group; ††*P*<0.01 vs. the HFD group; ^‡^ *P*<0.05, ^‡‡^ *P*<0.01 *vs.* the HFD+STZ group. CON: control; HFD: high-fat diet; CE: chronic exercise; STZ: streptozotocin. N = 7–8.

To verify that the increase in exercise-stimulated glucose uptake was related to changes of the AMPK pathway signalling in this setting, we measured phosphorylation and expression of AMPKα1/2, AMPKα1, AMPKα2, LKB1 and ACC in muscle lysates. Phosphorylation of AMPKα1/2, AMPKα1, AMPKα2 and LKB1 in the muscle was reduced (*P = *0.025, *P = *0.013, *P = *0.001 and *P = *0.016; Fig. 1*A, B, C and E*) in the HFD+STZ group compared with the control group. However, the changes in phosphorylation were increased by chronic exercise. These changes were accompanied by a similar trend in protein expression of AMPKα1/2, AMPKα1, AMPKα2 and LKB1 (Fig. 1*F*). By contrast, the phosphorylation and expression of AMPK downstream target, ACC, were significantly greater in the HFD+STZ group compared with the control group (Fig. 1*D and F*); these changes were suppressed by chronic exercise. Moreover, the results showed an increased ACC phosphorylation and a decreased AMPKα2 protein expression in the HFD+STZ group versus the HFD group (*P* = 0.001 and *P = *0.009; Fig. 1*D and F*). There was no significant effect of exercise on ACC phosphorylation and AMPKα2 protein expression in HFD rats, whereas exercise improved these changes in HFD+STZ rats as described above. Chronic exercise also increased the phosphorylation and expression of AMPKα1/2(Fig. 1*A and F*), AMPKα1(Fig. 1*B and F*), as well as LKB1 phosphorylation (Fig. 1*E*) in the HFD rats.

### Chronic Exercise Increased Phosphorylation and Expression of aPKC

**Figure 2:**
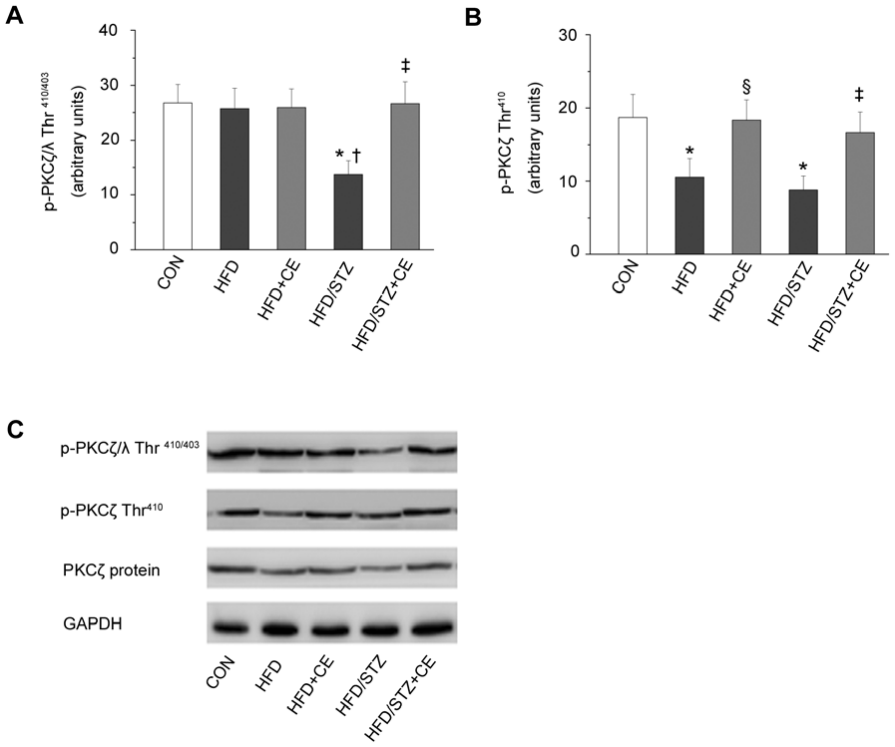
Effects of chronic exercise on the phosphorylation and protein expression of aPKC. p-PKCζ/λ (Thr^410/403^) (A) and p-PKCζ (Thr^410^) (B). C: representative western blots for each protein of interest. Values are means ± SE. ^*^ *P*<0.05 *vs.* the control group; ^§^ *P*<0.05 *vs.* the HFD group; †*P*<0.05 vs. the HFD group; ^‡^ *P*<0.05 *vs.* the HFD+STZ group. CON: control; HFD: high-fat diet; CE: chronic exercise; STZ: streptozotocin. N = 7–8.

It is unknown whether the effects of chronic exercise on aPKC expression and phosphorylation in HFD+STZ rats. Our results showed phosphorylation of PKCζ/λ, and phosphorylation and protein expression of PKCζ in the muscle were significantly lower in the HFD+STZ group than in the control group (*P = *0.011, *P = *0.013, and *P = *0.002; Fig. 2*A, B and C*). Chronic exercise improved the changes. Besides, the HFD significantly reduced the phosphorylation of PKCζ (*P* = 0.039), but exercise increased PKCζ phosphorylation (*P* = 0.047; Fig. 2*B*). Meanwhile, the phosphorylation of PKCζ/λ was marked decreased (*P* = 0.019; Fig. 2*A*) in the HFD+STZ rats compared with the HFD rats. However, the effects of exercise on phosphorylation of PKCζ/λ were only observed in the HFD+STZ rats.

### Effects of Chronic Exercise on Components of the Insulin Signaling Pathway

**Figure 3:**
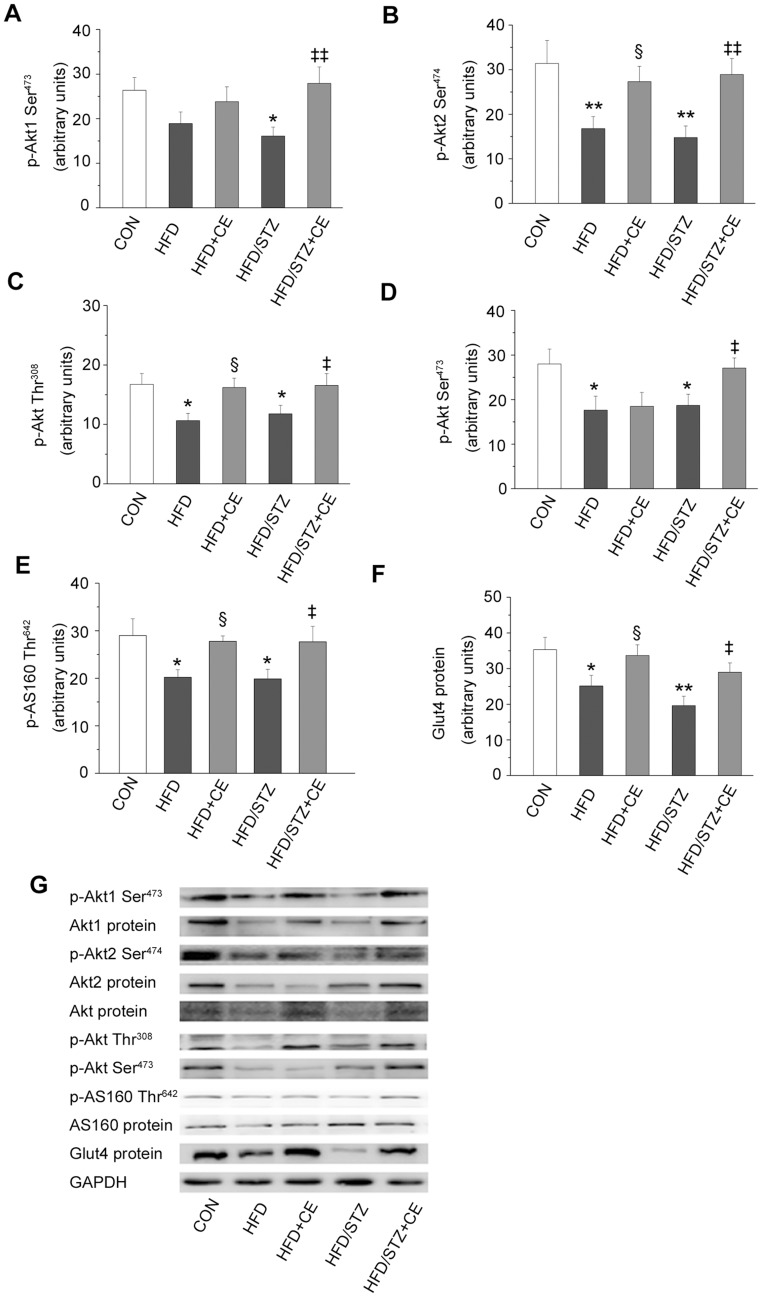
Effects of chronic exercise on components of the insulin signaling pathway. A–F: expression levels of p-Akt1 (Ser^473^) (A), p-Akt2 (Ser^474^) (B), p-Akt (Thr^308^) (C), p-Akt (Ser^473^) (D), p-AS160 (Thr^642^) (E), and GLUT4 (F). G: representative western blots of the proteins of interest. Values are shown as mean ± SE.^ *^ *P*<0.05 and ^**^ *P*<0.01 *vs.* the control group; ^§^ *P*<0.05 *vs.* the HFD group; ^‡‡^ *P*<0.01 *vs.* the HFD+STZ group. CON: control; HFD: high-fat diet; CE: chronic exercise; STZ: streptozotocin. N = 7–8.

To establish the impairments of the insulin signaling pathway and the effects of chronic exercise, we examined downstream components of PI3K, including phosphorylation and protein expression of Akt1, Akt2 and AS160, phosphorylation of Akt (Thr^308^) and Akt (Ser^ 473^), protein expression of Akt and GLUT4 both in HFD+STZ and HFD rats. Compared with the control group, phosphorylation of Akt1, Akt2, Akt (Thr^308^), Akt (Ser^ 473^) and AS160 in the muscle was reduced (*P = *0.019, *P = *0.003, *P = *0.04, *P = *0.029, *P = *0.016; Fig. 3*A, B, C, D and E*) in the HFD+STZ group. Additionally, we observed the muscle protein content of Akt1 (*P = *0.026; Fig.3G), Akt2(*P = *0.025; Fig.3G) and Akt (*P = *0.002; Fig.3G) was lower in the same group than in the control group. Chronic exercise improved the changes. Meanwhile, we also analyzed the protein expression of AS160 and GLUT4. In the line with the effects on Akt protein expression, exercise increased protein content of GLUT4 (*P = *0.032; Fig. 3*F*), but AS160 protein expression was unchanged (Fig. 3*G*) in the HFD+STZ rats. In addition, chronic exercise increased the phosphorylation of Akt2 and Akt (Thr^308^) and AS160 (Fig. 3*B, C and E*), as well as protein content of Akt1, Akt and GLUT4 in the HFD rats.

### Effects of Acute Exercise on Metabolic Parameters

Blood glucose concentrations measured at 8–16 h after acute exercise in the HFD+STZ+AE group were significantly lower than those in the HFD+STZ group. However, acute exercise did not improve body mass, or blood triglyceride, total cholesterol, free fatty acid, or insulin concentrations. Acute exercise increased the glucose disappearance rate in the HFD+STZ+AE group, although this was still lower than that in the control group (Table 1 and 2).

**Table 2:** Metabolic characteristics.

Item	HFD+STZ	HFD+STZ+AE
**Body mass, g**	502.8±10.51	507.3±13.16
**Blood glucose, mmol/L**	13.18±0.52	11.51±0.416^‡^
**Insulin, pmol/L**	76.29±4.17	68.70±3.34
**Triglyceride, mmol/L**	2.06±0.11	2.12±0.079
**Total cholesterol, mmol/L**	5.78±0.39	5.67±0.35
**Free fatty acid, mmol/L**	0.63±0.05	0.60±0.04
***K*** **_itt_, %/min**	1.95±0.30	2.61±0.15^‡^
**3MG uptake,µmol/g/h**	0.059±0.007	0.077±0.004^‡^
**Muscle glycogen, µmol/g wet weight**	22.50±0.23	23.09±0.30

Values are means ± SE. ^‡^ *P*<0.05 *vs.* the HFD+STZ group. HFD: high-fat diet; STZ: streptozotocin; AE: acute exercise. N = 8.

3MG uptake was reduced by 55% in the HFD+STZ group compared with the control group, but was increased by 30% at 16 h after acute exercise in the HFD+STZ+AE group (*P*<0.05), indicating that acute exercise does not completely compensate for muscle insulin resistance in HFD+STZ rats. Although acute exercise increased glycogen content in the HFD+STZ+AE group compared with the HFD+STZ group, this difference was not statistically significant (Table 2).

### Acute Exercise Improves AMPK Pathway in HFD+STZ Rats

**Figure 4:**
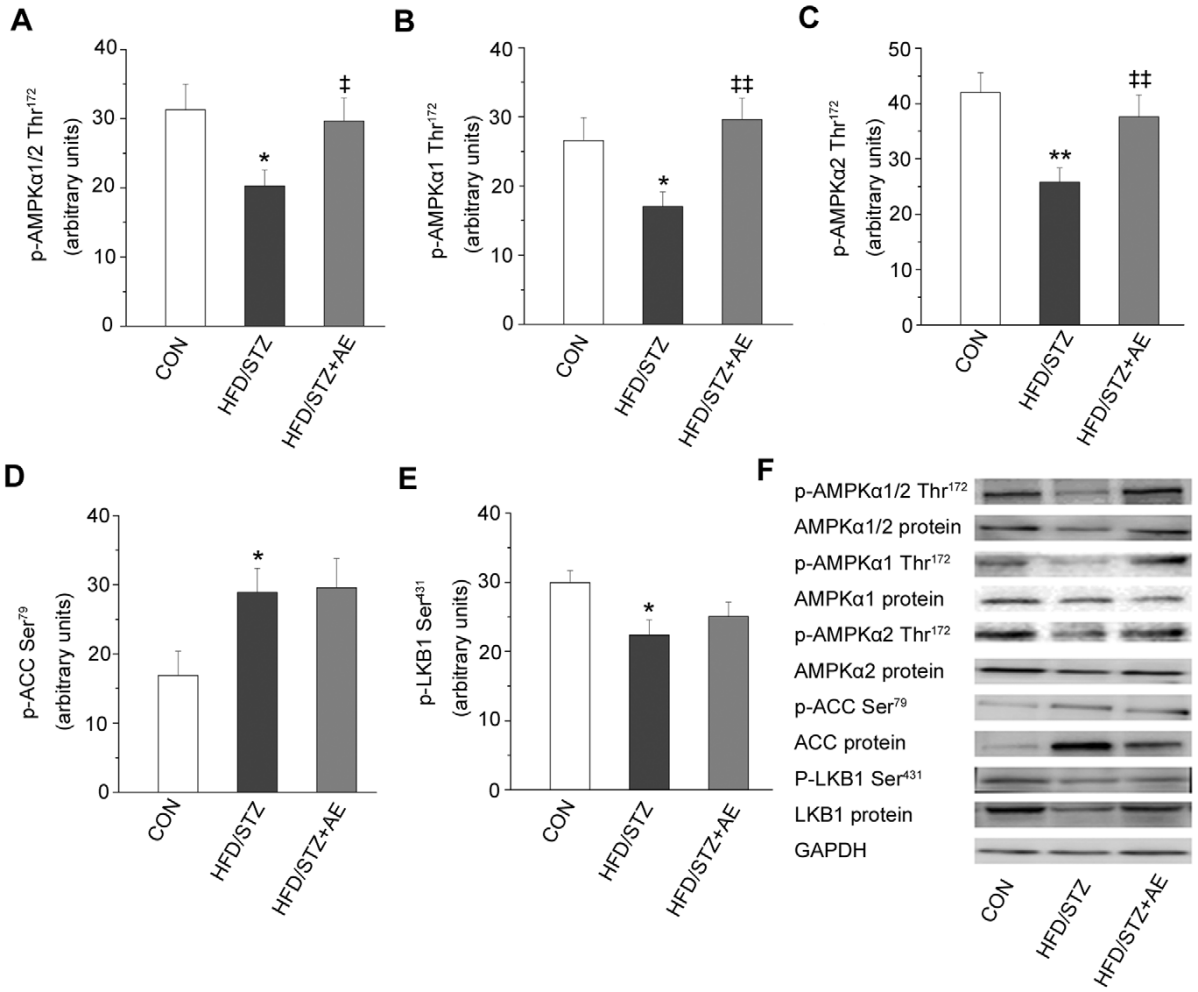
Effects of acute exercise on the protein kinases in the AMPK pathway. A–E: relative expression levels of p-AMPKα1/2 (Thr^172^) (A), p-AMPKα1 (Thr^172^) (B), p-AMPKα2 (Thr^172^) (C), p-ACC (Ser^79^) (D), and p-LKB1 (Ser^431^) (E). F: representative western blots for each protein of interest. Values are means ± SE. ^*^ *P*<0.05 and ^**^ *P*<0.01 *vs.* the control group; ^‡^ *P*<0.05 and ^‡‡^ *P*<0.01 *vs.* the HFD+STZ group. CON: control; HFD: high-fat diet; STZ: streptozotocin; AE: acute exercise. N = 7–8.

We also examined the effect of acute exercise on the AMPK pathway at different signaling levels. Compared with the HFD+STZ group, acute exercise increased AMPKα1/2 phosphorylation and protein content (*P = *0.049 and *P = *0.030, respectively; Fig. 4*A and F*). Acute exercise also reversed the effects of HFD+STZ on AMPKα1 phosphorylation, and AMPKα2 phosphorylation and protein expression (*P* = 0.004, *P = *0.024, and *P = *0.009, respectively; Fig. 4*B, C and F*), but not AMPKα1 protein expression (Fig. 4*F*). Acute exercise also decreased ACC protein expression (*P* = 0.008; Fig. 4*F*), but not ACC phosphorylation (Fig. 4*D*). Moreover, acute exercise increased LKB1 protein expression (*P = *0.018; Fig. 4*F*).

### Acute Exercise Reversed the Effects of HFD+STZ on the Phosphorylation and Expression of aPKC

**Figure 5:**
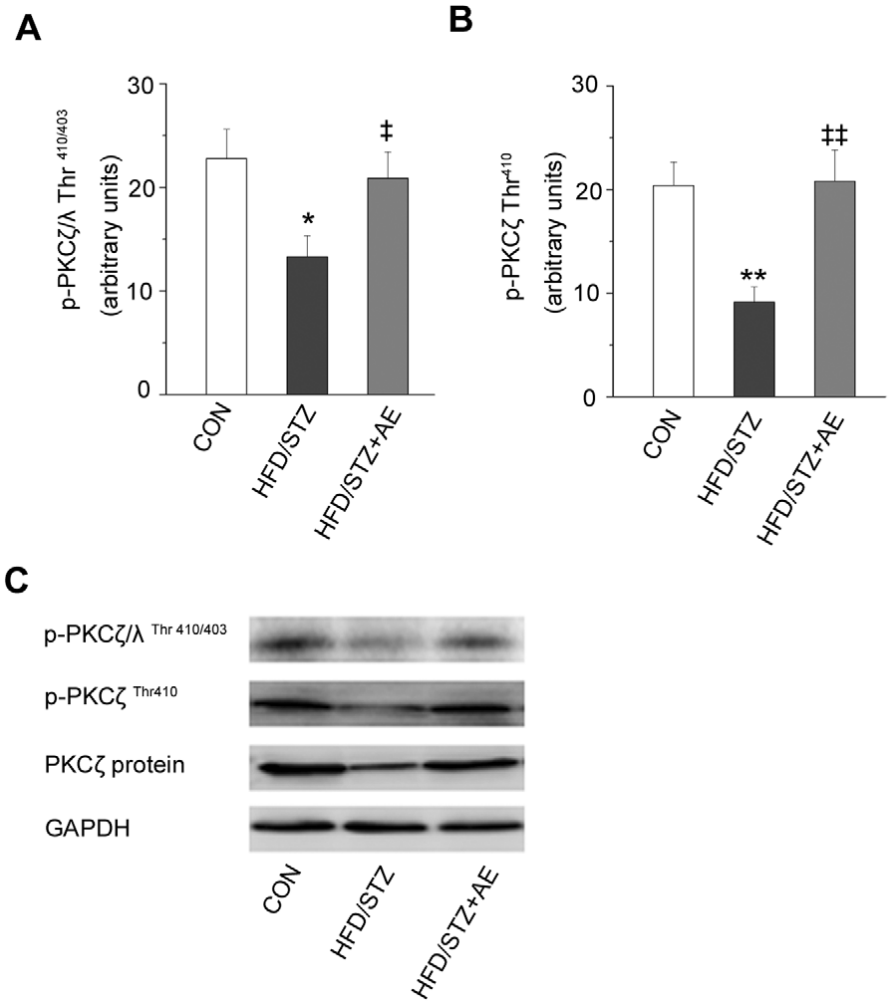
Effects of acute exercise on the phosphorylation and protein expression of aPKC. p-PKCζ/λ (Thr^410/403^) (A) and p-PKCζ (Thr^410^) (B). C: representative western blots for each protein of interest. Values are means ± SE. ^*^ *P*<0.05 and^ **^ *P*<0.01 *vs.* the control group; ^‡^ *P*<0.05, ^‡‡^ *P*<0.01 *vs.* the HFD+STZ group. CON: control; HFD: high-fat diet; STZ: streptozotocin; AE: acute exercise. N = 7–8.

It has not been observed whether acute exercise influences the phosphorylation and expression of aPKC in HFD+STZ rats. Our data showed acute exercise increased the phosphorylation of PKCζ/λ after the acute exercise, as compared with the HFD+STZ group (*P* = 0.043; Fig. 5*A*). Moreover, PKCζ phosphorylation and protein expression were significantly upregulated in the HFD+STZ+AE group compared with the HFD+STZ group (*P = *0.002 and *P = *0.003, respectively; Fig. 5*B and C*).

### Effects of Acute Exercise on Components of the Insulin Signaling Pathway in Muscle in HFD+STZ rats

**Figure 6:**
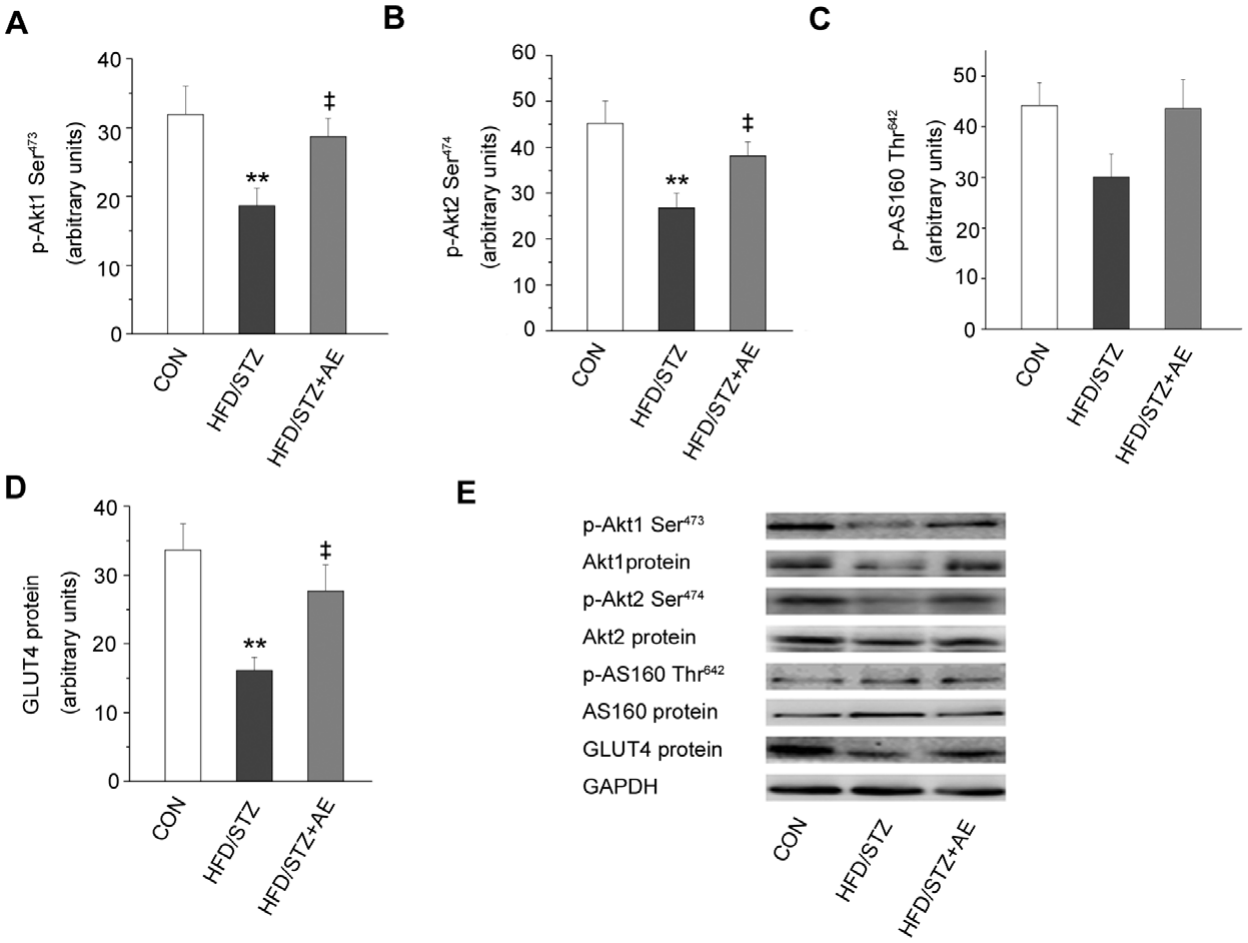
Effects of acute exercise on components of the insulin signaling pathway. A–F: expression levels of p-Akt1 (Ser^473^) (A), p-Akt2 (Ser^474^) (B), p-AS160 (Thr^642^) (C), and GLUT4 (D). E: representative western blots of the proteins of interest. Values are means ± SE. ^**^ *P*<0.01 *vs.* the control group; ^‡^ *P*<0.05 and ^‡‡^ *P*<0.01 *vs.* the HFD+STZ group. CON: control; HFD: high-fat diet; STZ: streptozotocin; AE: acute exercise. N = 7–8.

To investigate skeletal muscle responses to acute exercise, we analyzed downstream components of PI3K insulin signaling. Acute exercise enhanced Akt1 phosphorylation and protein expression (*P = *0.038 and *P = *0.041, respectively; Fig. 6*A and E*). Acute exercise also increased Akt2 phosphorylation (*P = *0.049; Fig. 6*B*), but not Akt2 protein expression (Fig. 6*E*). AS160 phosphorylation only tended to increase after the acute exercise in the HFD+STZ group (Fig. 6*C*). Besides, acute exercise also significantly increased GLUT4 protein expression in the HFD+STZ group (*P = *0.022; Fig. 6*D*).

### Effects of Chronic and Acute Exercise on *PGC1*α and *NRF1* mRNA Expression

**Figure 7:**
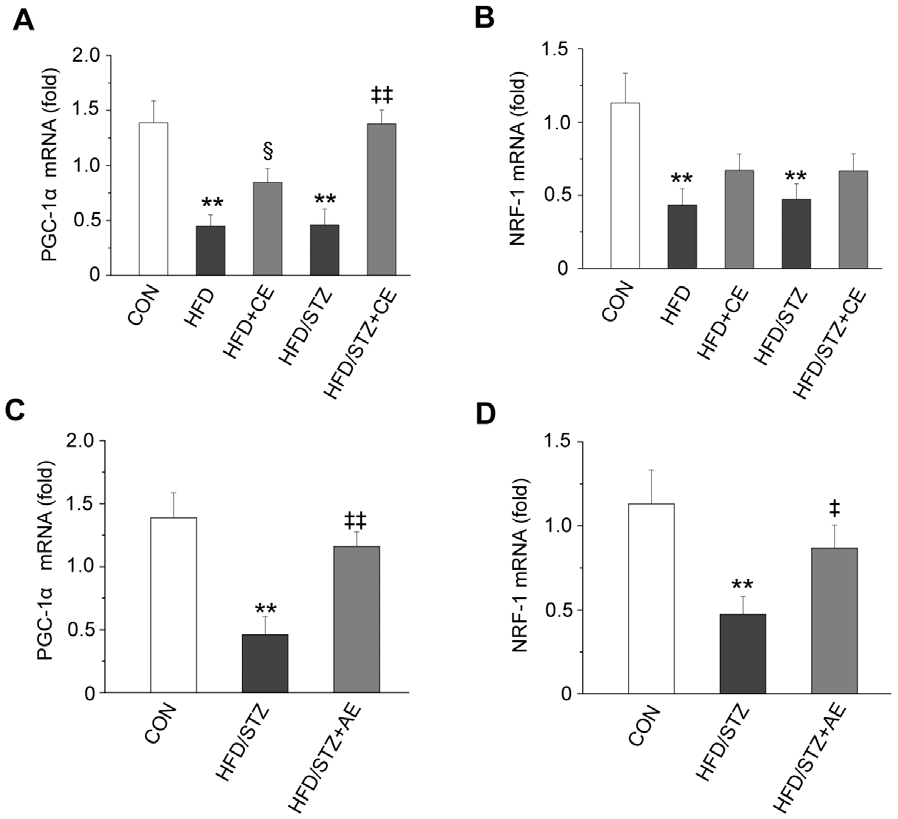
Effects of chronic and acute exercise on *PGC1α* and *NRF1* mRNA expression. A,B: effects of chronic exercise. C,D: effects of acute exercise. Values are means ± SE. ^**^ *P*<0.01 *vs.* the control group; ^§^ *P*<0.05 *vs.* the HFD+STZ group; ^‡^ *P*<0.05 and ^‡‡^ *P*<0.01 *vs.* the HFD+STZ group. *PGC1α*: peroxisome proliferator-activated receptor-γ coactivator; *NRF1*: nuclear respiratory factor 1; CON: control; HFD: high-fat diet; STZ, streptozotocin; CE: chronic exercise; AE: acute exercise. N = 8.

We evaluated the effects of exercise on *PGC1α* and *NRF1* gene expression in the HFD and HFD+STZ groups. *PGC1α* mRNA expression in the muscle was significantly lower in the HFD and HFD+STZ groups compared with the control group (*P*<0.001 and *P*<0.001, respectively; Fig. 7*A*). Chronic exercise increased *PGC1α* mRNA expression in the HFD+CE and HFD+STZ+CE groups (*P = *0.048 and *P*<0.001, respectively). *NRF1* mRNA expression was also significantly reduced in the HFD and HFD+STZ groups compared with the control group (*P* = 0.001 and *P* = 0.001, respectively; Fig. 7*B*). Although chronic exercise increased *NRF*1 mRNA expression in the HFD+CE and HFD+STZ +CE groups, the difference had no statistical significance. Acute exercise in the HFD+STZ+AE group significantly increased the mRNA expression of both *PGC1α* and *NRF1* compared with the HFD+STZ group (*P = *0.001 and *P = *0.045; Fig. 7*C and D*).

## Discussion

### Summary of Findings

The results of this study showed that the pathogenesis and metabolic characteristics of rats with HFD+STZ-induced type 2 diabetes were similar to those in humans. Chronic and acute exercise improved phosphorylation and protein expression levels of components of the skeletal muscle AMPK pathway, as well as the phosphorylation and expression of aPKC, Akt and Akt isoforms. Together, these changes conferred enhanced GLUT4 translocation to membrane and promoted 3MG uptake in these rats. Chronic and acute exercise also reversed the downregulation of *PGC1α* mRNA caused by HFD+STZ.

### Mechanisms of Chronic Exercise-stimulated Glucose Uptake

#### Changes of AMPK signaling

AMPK plays a key role in fat and carbohydrate metabolism. Presumably, defects in AMPK signaling could somehow result in the metabolic abnormalities of type 2 diabetes [35]. It has become apparent that AMPK was important in mediating the acute and chronic effects of exercise on glucose and lipid metabolism in skeletal muscle [36], [37]. However, under insulin-resistant conditions, the results of studies of food-induced or genetic obesity differed. Skeletal muscle AMPK phosphorylation was reduced in HFD-induced obese Wistar rats and in obese Zucker rats [35], [38]. Acute exercise was reported to increase AMPK phosphorylation in Wistar rats [38], but not in obese Zucker rats [35]. AMPK phosphorylation and protein expression were not adversely affected in the skeletal muscle of *ob/ob* mice and obese Wistar rats with insulin resistance induced by cafeteria diet [39], [40]. However, exercise enhanced AMPKα2 expression in Wistar rats above [39]. These differing results may be due to differences in the species of the experimental animals. Interestingly, we found marked differences between the HFD and HFD+STZ groups in this study. In particular, the HFD+STZ group showed altered AMPKα1 and α2 expression, AMPKα1 and α2Thr^172^ phosphorylation and ACC expression and phosphorylation, all of which were improved by exercise. A previous study demonstrated that exercise increased AMPKα2 expression and AMPK Thr^172^ phosphorylation in diabetic patients [41]. In the present study, AMPKα1 expression and Thr^172^ phosphorylation were reduced in HFD-induced obese and insulin-resistant rats, which were reversed by chronic exercise, consistent with previous results [4]. Although the mechanisms responsible for the different effects of obesity and insulin resistance, especially in type 2 diabetes, on AMPK signaling components are unclear, it is apparent that chronic exercise increase AMPK expression and downregulate expression and phosphorylation of its downstream target, ACC. These effects of exercise are partly associated with enhanced glucose transport [36] and lipid oxidation [37] at least.

#### Findings in aPKC

The characteristics of aPKC, which lie downstream of PI3K, have been described in recent years. In particular, it has been reported that aPKC plays a key role in normal insulin-stimulated glucose uptake [42]. Several studies have shown that insulin stimulation and exercise can increase skeletal muscle aPKC activity [23], [42], [43], [44]. However, glucose absorption capacity is more strongly induced by insulin-mediated aPKC activity than by exercise-mediated signaling [23], [44]. HFD-induced insulin resistance in rats reduced insulin’s ability to stimulate PKCζ/λ activity, even though the phosphorylation and protein expression levels of PKCζ and PKCλ remained unchanged [26], [45], [46]. In the present study, HFD+STZ reduced PKCζ/λ phosphorylation, as well as PKCζ phosphorylation and expression in skeletal muscle, effects that were reversed by chronic exercise. Kanzaki *et al* [47] reported that PKCζ/λ interacts with vesicles containing GLUT4. PKCζ regulates munc18, a protein that facilitates the transport of vesicles containing GLUT4 [48]. Therefore, exercise may enhance the activity of PKCζ/λ to aid GLUT4 translocation. However, downstream signaling of aPKC that promote translocation of GLUT4 vesicles are still unknown, and the precise mechanism by which aPKC regulate GLUT4 translocation is now being explored [42], [43].

#### Downstream components of PI3K

Akt, AS160, and GLUT4, are all components of the classical insulin pathway. Chronic exercise increased the phosphorylation and protein expression of the Akt1 and 2 isoforms in the HFD+STZ group, consistent with the responses of Akt (Ser^473^) and Akt (Thr^308^) phosphorylation to exercise. However, a previous study showed that exercise only restored Akt1 expression in rats with HFD-induced insulin resistance, and Akt2 content was unchanged [4]. Akt is the key component of the insulin signaling pathway, and promotes glucose transport via AS160 [49]. AS160 was the first component of the insulin signaling pathway found to directly promote GLUT4 membrane transport [42]. A recent study has confirmed the effects of AS160 in inducing glucose absorption in skeletal muscle [50]. In the present study, we found the phosphorylation of AS160 and GLUT4 expression were increased in the HFD+CE and HFD+STZ+CE groups. Therefore, the results from the present study indicate that exercise-induced activation of the Akt–AS160–GLUT4 pathway is important for improving skeletal muscle glucose handling in HFD rats and HFD+STZ-induced diabetic rats.

#### AMPK upstream kinase, LKB1

Experiments using mice with muscle-specific LKB1 knockout showed that LKB1 is a major kinase upstream of AMPK involved in the contractile response, and is an important regulator of skeletal muscle glucose transport [17], [51]. However, Sriwijitkamol *et al* [52] reported that exercise did not affect LKB1 content or activity in obese and type 2 diabetic patients. In the present study, chronic exercise improved LKB1 content and phosphorylation, which were otherwise reduced in the HFD and HFD+STZ groups. An earlier study showed that skeletal muscle LKB1 expression was reduced in obese and insulin-resistant Zucker rats, which was reversed by chronic exercise [35]. The differences in results among these studies may be due to differences in exercise pattern and/or intensity.

### Mechanisms of Acute Exercise-stimulated Glucose Uptake

Acute exercise significantly enhanced insulin’s ability to stimulate glucose absorption in skeletal muscle and counteracted insulin resistance, a typical feature of type 2 diabetes. However, the potential molecular mechanism remains poorly understood [42]. Therefore, in this study we subjected HFD+STZ-induced diabetic rats to a session of acute exercise. We found that acute exercise improved the phosphorylation and protein expression of the protein kinase cascade in the AMPK signaling pathway. Interestingly, acute exercise reversed the effects of HFD+STZ on aPKC phosphorylation and expression, increased Akt subunit phosphorylation and expression, elevated GLUT4 content, and enhanced glucose absorption in skeletal muscle. However, there were some notable differences in the characteristics of rats subjected to chronic and acute exercise. For example, blood triglyceride, total cholesterol, and free fatty acid concentrations were not affected by acute exercise. The main difference in signaling pathways between chronic and acute exercise was related to phosphorylation of ACC and LKB1, and AKT2 protein content, which did not change after an acute bout of exercise. It has been postulated that the discrepancy may reflect the influence of different modes of chronic and acute exercise, as well as exercise intensity. Given this contention, it is also plausible that differences in the signaling response between models could be related to the time points chosen for evaluation. However, the net effects of acute and chronic exercise on the AMPK and downstream to PIK3 signaling pathways were broadly comparable.

### Gene Expression of *PGC1α* and *NRF1* Response to Exercise

Exercise improves skeletal muscle AMPK activity, and enhances mitochondrial biogenesis and function [35], [53]. An increase in mitochondrial quantity and function is thought to involve activation of the *PGC1α* gene by the AMPK-mediated peroxisome proliferator [54]. Therefore, AMPK may directly contribute to the exercise-induced increase in *PGC1α* gene expression. The results of this study suggest that by enhancing AMPK activity, chronic and acute exercise reversed the downregulation of gene expression in the HFD+STZ rats. As a transcriptional coactivator, PGC-1 is a major coordinator of mitochondrial biogenesis, and acts by interacting with NRF-1 [55]. Thus, we also assessed *NRF1* gene expression in this study. We found that acute exercise restored the reduction in *NRF1* gene expression induced by HFD+STZ in muscle.

### Conclusion

In summary, chronic and acute exercise improved skeletal muscle AMPK signaling and downstream to PIK3 in rats with HFD- plus STZ-induced diabetes. Although the HFD adversely affected the phosphorylation and expression of several components of the AMPK signaling and downstream to PIK3, chronic exercise improved these changes. The present study provides important insights into the mechanisms by which exercise may correct the molecular disorders associated with obesity and type 2 diabetes.

## References

[1] Højlund K, Mogensen M, Sahlin K, Beck-Nielsen H (2008) Mitochondrial dysfunction in type 2 diabetes and obesity. Endocrinol Metab Clin North Am 37: 713–731, x. 10.1016/j.ecl.2008.06.006 18775360

[2] Kim CH, Youn JH, Park JY, Hong SK, Park KS, et al (2000) Effects of high-fat diet and exercise training on intracellular glucose metabolism in rats. Am J Physiol Endocrinol Metab 278: E977–984. 10826998 10.1152/ajpendo.2000.278.6.E977

[3] Kraegen EW, Storlien LH, Jenkins AB, James DE (1989) Chronic exercise compensates for insulin resistance induced by a high-fat diet in rats. Am J Physiol 256: E242–249. 2645785 10.1152/ajpendo.1989.256.2.E242

[4] Lessard SJ, Rivas DA, Chen ZP, Bonen A, Febbraio MA, et al (2007) Tissue-specific effects of rosiglitazone and exercise in the treatment of lipid-induced insulin resistance. Diabetes 56: 1856–1864. 17440174 10.2337/db06-1065

[5] Srinivasan K, Viswanad B, Asrat L, Kaul CL, Ramarao P (2005) Combination of high-fat diet-fed and low-dose streptozotocin-treated rat: a model for type 2 diabetes and pharmacological screening. Pharmacol Res 52: 313–320. 15979893 10.1016/j.phrs.2005.05.004

[6] Reed MJ, Meszaros K, Entes LJ, Claypool MD, Pinkett JG, et al (2000) A new rat model of type 2 diabetes: the fat-fed, streptozotocin-treated rat. Metabolism 49: 1390–1394. 11092499 10.1053/meta.2000.17721

[7] Zhang M, Lv XY, Li J, Xu ZG, Chen L (2008) The Characterization of High-Fat Diet and Multiple Low-Dose Streptozotocin Induced Type 2 Diabetes Rat Model. Exp Diabetes Res 2008: 704045. 19132099 10.1155/2008/704045 PMC2613511

[8] Musi N, Hayashi T, Fujii N, Hirshman MF, Witters LA, et al (2001) AMP-activated protein kinase activity and glucose uptake in rat skeletal muscle. Am J Physiol Endocrinol Metab 280: E677–684. 11287349 10.1152/ajpendo.2001.280.5.E677

[9] Ye JM, Dzamko N, Hoy AJ, Iglesias MA, Kemp B, et al (2006) Rosiglitazone treatment enhances acute AMP-activated protein kinase-mediated muscle and adipose tissue glucose uptake in high-fat-fed rats. Diabetes 55: 2797–2804. 17003345 10.2337/db05-1315

[10] Fujii N, Ho RC, Manabe Y, Jessen N, Toyoda T, et al (2008) Ablation of AMP-activated protein kinase alpha2 activity exacerbates insulin resistance induced by high-fat feeding of mice. Diabetes 57: 2958–2966. 18728234 10.2337/db07-1187 PMC2570392

[11] Yu X, McCorkle S, Wang M, Lee Y, Li J, et al (2004) Leptinomimetic effects of the AMP kinase activator AICAR in leptin-resistant rats: prevention of diabetes and ectopic lipid deposition. Diabetologia 47: 2012–2021. 15578153 10.1007/s00125-004-1570-9

[12] Richter EA, Ruderman NB (2009) AMPK and the biochemistry of exercise: implications for human health and disease. Biochem J 418: 261–275. 19196246 10.1042/BJ20082055 PMC2779044

[13] Hawley JA, Lessard SJ (2008) Exercise training-induced improvements in insulin action. Acta Physiol (Oxf) 192: 127–135. 18171435 10.1111/j.1748-1716.2007.01783.x

[14] Wojtaszewski JF, Richter EA (2006) Effects of acute exercise and training on insulin action and sensitivity: focus on molecular mechanisms in muscle. Essays Biochem 42: 31–46. 17144878 10.1042/bse0420031

[15] Fujii N, Hirshman MF, Kane EM, Ho RC, Peter LE, et al (2005) AMP-activated protein kinase α2 activity is not essential for contraction- and hyperosmolarity-induced glucose transport in skeletal muscle. J Biol Chem 280: 39033–39041. 16186119 10.1074/jbc.M504208200

[16] Jørgensen SB, Viollet B, Andreelli F, Frøsig C, Birk JB, et al (2004) Knockout of the alpha2 but not alpha1 5′-AMP-activated protein kinase isoform abolishes 5-aminoimidazole-4-carboxamide-1-beta-4-ribofuranosidebut not contraction-induced glucose uptake in skeletal muscle. J Biol Chem 279: 1070–1079. 14573616 10.1074/jbc.M306205200

[17] Sakamoto K, McCarthy A, Smith D, Green KA, Grahame Hardie D, et al (2005) Deficiency of LKB1 in skeletal muscle prevents AMPK activation and glucose uptake during contraction. EMBO J 24: 1810–1820. 15889149 10.1038/sj.emboj.7600667 PMC1142598

[18] Goodyear LJ, Kahn BB (1998) Exercise, glucose transport, and insulin sensitivity. Annu Rev Med 49: 235–261. 9509261 10.1146/annurev.med.49.1.235

[19] Hayashi T, Wojtaszewski JF, Goodyear LJ (1997) Exercise regulation of glucose transport in skeletal muscle. Am J Physiol 273: E1039–1051. 9435517 10.1152/ajpendo.1997.273.6.E1039

[20] Folli F, Saad MJ, Backer JM, Kahn CR (1992) Insulin stimulation of phosphatidylinositol 3-kinase activity and association with insulin receptor substrate 1 in liver and muscle of the intact rat. J Biol Chem 267: 22171–22177. 1385396

[21] Goodyear LJ, Giorgino F, Balon TW, Condorelli G, Smith RJ (1995) Effects of contractile activity on tyrosine phosphoproteins and phosphatidylinositol 3-kinase activity in rat skeletal muscle. Am J Physiol 268: E987–995. 7762655 10.1152/ajpendo.1995.268.5.E987

[22] Treadway JL, James DE, Burcel E, Ruderman NB (1989) Effect of exercise on insulin receptor binding and kinase activity in skeletal muscle. Am J Physiol 256: E138–144. 2643337 10.1152/ajpendo.1989.256.1.E138

[23] Farese RV, Sajan MP, Yang H, Li P, Mastorides S, et al (2007) Musclespecific knockout of PKC-lambda impairs glucose transport and induces metabolic and diabetic syndromes. J Clin Invest 117: 2289–2301. 17641777 10.1172/JCI31408 PMC1913489

[24] Richter EA, Vistisen B, Maarbjerg SJ, Sajan M, Farese RV, et al (2004) Differential effect of bicycling exercise intensity on activity and phosphorylation of atypical protein kinase C and extracellular signal-regulated protein kinase in skeletal muscle. J Physiol 560: 909–918. 15297577 10.1113/jphysiol.2004.071373 PMC1665296

[25] Braiman L, Alt A, Kuroki T, Ohba M, Bak A, et al (2001) Activation of protein kinase C zeta induces serine phosphorylation of VAMP2 in the GLUT4 compartment and increases glucose transport in skeletal muscle. Mol Cell Biol 21: 7852–7861. 11604519 10.1128/MCB.21.22.7852-7861.2001 PMC99955

[26] Yaspelkis BB 3rd, Lessard SJ, Reeder DW, Limon JJ, Saito M, et al (2007) Exercise reverses high-fat diet-induced impairments on compartmentalization and activation of components of the insulin-signaling cascade in skeletal muscle. Am J Physiol Endocrinol Metab 293: E941–949. 17623749 10.1152/ajpendo.00230.2007

[27] Lavigne C, Tremblay F, Asselin G, Jacques H, Marette A (2001) Prevention of skeletal muscle insulin resistance by dietary cod protein in high fat-fed rats. Am J Physiol Endocrinol Metab 281: E62–71. 11404223 10.1152/ajpendo.2001.281.1.E62

[28] Luciano E, Carneiro EM, Carvalho CR, Carvalheira JB, Peres SB, et al (2002) Endurance training improves responsiveness to insulin and modulates insulin signal transduction through the phosphatidylinositol 3-kinase/Akt-1 pathway. Eur J Endocrinol 147: 149–157. 12088932 10.1530/eje.0.1470149

[29] Chibalin AV, Yu M, Ryder JW, Song XM, Galuska D, et al (2000) Exercise-induced changes in expression and activity of proteins involved in insulin signal transduction in skeletal muscle: differential effects on insulin-receptor substrates 1 and 2. Proc Natl Acad Sci USA 97: 38–43. 10618367 10.1073/pnas.97.1.38 PMC26612

[30] Scott AM, Atwater I, Rojas E (1981) A method for the simultaneous measurement of insulin release and B cell membrane potential in single mouse islets of Langerhans. Diabetologia 21: 470–475. 7028561 10.1007/BF00257788

[31] Bonora E, Moghetti P, Zancanaro C, Cigolini M, Querena M, et al (1989) Estimates of in vivo insulin action in man: comparison of insulin tolerance tests with euglycemic and hyperglycemic glucose clamp studies. J Clin Endocrinol Metab 68: 374–378. 2645308 10.1210/jcem-68-2-374

[32] Hayashi T, Hirshman MF, Kurth EJ, Winder WW, Goodyear LJ (1998) Evidence for 5′ AMP-activated protein kinase mediation of the effect of muscle contraction on glucose transport. Diabetes 47: 1369–1373. 9703344 10.2337/diab.47.8.1369

[33] Tanaka S, Hayashi T, Toyoda T, Hamada T, Shimizu Y, et al (2007) High-fat diet impairs the effects of a single bout of endurance exercise on glucose transport and insulin sensitivity in rat skeletal muscle. Metabolism 56: 1719–1728. 17998027 10.1016/j.metabol.2007.07.017

[34] Laemmli UK (1970) Cleavage of structural proteins during the assembly of the head of bacteriophage T4. Nature 227: 680–685. 5432063 10.1038/227680a0

[35] Sriwijitkamol A, Ivy JL, Christ-Roberts C, DeFronzo RA, Mandarino LJ, et al (2006) LKB1-AMPK signaling in muscle from obese insulin-resistant Zucker rats and effects of training. Am J Physiol Endocrinol Metab 290: E925–932. 16352671 10.1152/ajpendo.00429.2005

[36] Musi N, Fujii N, Hirshman MF, Ekberg I, Fröberg S, et al (2001) AMP-activated protein kinase (AMPK) is activated in muscle of subjects with type 2 diabetes during exercise. Diabetes 50: 921–927. 11334434 10.2337/diabetes.50.5.921

[37] Winder WW, Hardie DG (1996) Inactivation of acetyl-CoA carboxylase and activation of AMP-activated protein kinase in muscle during exercise. Am J Physiol Endocrinol Metab 270: E299–304. 10.1152/ajpendo.1996.270.2.E299 8779952

[38] Pauli JR, Ropelle ER, Cintra DE, Carvalho-Filho MA, Moraes JC, et al (2008) Acute physical exercise reverses S-nitrosation of the insulin receptor, insulin receptor substrate 1 and protein kinase B/Akt in diet-induced obese Wistar rats. J Physiol 586: 659–671. 17974582 10.1113/jphysiol.2007.142414 PMC2375587

[39] Brandt N, De Bock K, Richter EA, Hespel P (2010) Cafeteria diet-induced insulin resistance is not associated with decreased insulin signaling or AMPK activity and is alleviated by physical training in rats. Am J Physiol Endocrinol Metab 299: E215–224. 20484011 10.1152/ajpendo.00098.2010

[40] Li L, Pan R, Li R, Niemann B, Aurich AC, et al (2011) Mitochondrial biogenesis and peroxisome proliferator-activated receptor-γ coactivator-1α (PGC-1α) deacetylation by physical activity: intact adipocytokine signaling is required. Diabetes 60: 157–167. 20929977 10.2337/db10-0331 PMC3012167

[41] Vind BF, Pehmøller C, Treebak JT, Birk JB, Hey-Mogensen M, et al (2011) Impaired insulin-induced site-specific phosphorylation of TBC1 domain family, member 4 (TBC1D4) in skeletal muscle of type 2 diabetes patients is restored by endurance exercise-training. Diabetologia 54: 157–167. 20938636 10.1007/s00125-010-1924-4

[42] Frøsig C, Richter EA (2009) Improved insulin sensitivity after exercise: focus on insulin signaling. Obesity (Silver Spring) 17: S15–20. 19927140 10.1038/oby.2009.383

[43] Maarbjerg SJ, Sylow L, Richter EA (2011) Current understanding of increased insulin sensitivity after exercise - emerging candidates. Acta Physiol (Oxf) 202: 323–335. 21352505 10.1111/j.1748-1716.2011.02267.x

[44] Sajan MP, Bandyopadhyay G, Miura A, Standaert ML, Nimal S, et al (2010) AICAR and metformin, but not exercise, increase muscle glucose transport through AMPK-, ERK-, and PDK1-dependent activation of atypical PKC. Am J Physiol Endocrinol Metab 298: E179–192. 19887597 10.1152/ajpendo.00392.2009 PMC2822478

[45] Herr HJ, Bernard JR, Reeder DW, Rivas DA, Limon JJ, et al (2005) Insulin-stimulated plasma membrane association and activation of Akt2, aPKC zeta and aPKC lambda in high fat fed rodent skeletal muscle. J Physiol 565: 627–636. 15802290 10.1113/jphysiol.2005.086694 PMC1464539

[46] Kanoh Y, Sajan MP, Bandyopadhyay G, Miura A, Standaert ML, et al (2003) Defective activation of atypical protein kinase C zeta and lambda by insulin and phosphatidylinositol-3,4,5-(PO4)(3) in skeletal muscle of rats following high-fat feeding and streptozotocin-induced diabetes. Endocrinology 144: 947–954. 12586772 10.1210/en.2002-221017

[47] Kanzaki M, Mora S, Hwang JB, Saltiel AR, Pessin JE (2004) Atypical protein kinase C (PKCzeta/lambda) is a convergent downstream target of the insulin-stimulated phosphatidylinositol 3-kinase and TC10 signaling pathways. J Cell Biol 164: 279–290. 14734537 10.1083/jcb.200306152 PMC2172328

[48] Hodgkinson CP, Mander A, Sale GJ (2005) Protein kinase-zeta interacts with munc18c: role in GLUT4 trafficking. Diabetologia 48: 1627–1636. 15986239 10.1007/s00125-005-1819-y

[49] Taniguchi CM, Emanuelli B, Kahn CR (2006) Critical nodes in signalling pathways: insights into insulin action. Nat Rev Mol Cell Biol 7: 85–96. 16493415 10.1038/nrm1837

[50] Chen S, Wasserman DH, MacKintosh C, Sakamoto K (2011) Mice with AS160/TBC1D4-Thr649Ala knockin mutation are glucose intolerant with reduced insulin sensitivity and altered GLUT4 trafficking. Cell Metab 13: 68–79. 21195350 10.1016/j.cmet.2010.12.005 PMC3081066

[51] Koh HJ, Toyoda T, Fujii N, Jung MM, Rathod A, et al (2010) Sucrose nonfermenting AMPK-related kinase (SNARK) mediates contraction-stimulated glucose transport in mouse skeletal muscle. Proc Natl Acad Sci USA 107: 15541–15546. 20713714 10.1073/pnas.1008131107 PMC2932588

[52] Sriwijitkamol A, Coletta DK, Wajcberg E, Balbontin GB, Reyna SM, et al (2007) Effect of acute exercise on AMPK signaling in skeletal muscle of subjects with type 2 diabetes: a time-course and dose-response study. Diabetes 56: 836–848. 17327455 10.2337/db06-1119 PMC2844111

[53] Bergeron R, Ren JM, Cadman KS, Moore IK, Perret P, P et al (2001) Chronic activation of AMP kinase results in NRF-1 activation and mitochondrial biogenesis. Am J Physiol Endocrinol Metab 281: E1340–1346. 11701451 10.1152/ajpendo.2001.281.6.E1340

[54] Terada S, Goto M, Kato M, Kawanaka K, Shimokawa T, et al (2002) Effects of low-intensity prolonged exercise on PGC-1 mRNA expression in rat epitrochlearis muscle. Biochem Biophys Res Commun 296: 350–354. 12163024 10.1016/s0006-291x(02)00881-1

[55] Puigserver P, Spiegelman BM (2003) Peroxisome proliferator-activated receptor-gamma coactivator 1 alpha (PGC-1 alpha): transcriptional coactivator and metabolic regulator. Endocr Rev 24: 78–90. 12588810 10.1210/er.2002-0012

